# Supplementary figures and images for: Genome-Wide Association Mapping of Processing Quality Traits in Common Wheat (Triticum aestivum L.)
Source: Genes (Basel). 2023 Sep 18;14(9):1816. doi: 10.3390/genes14091816 (PMC10530800; doi:10.3390/genes14091816)

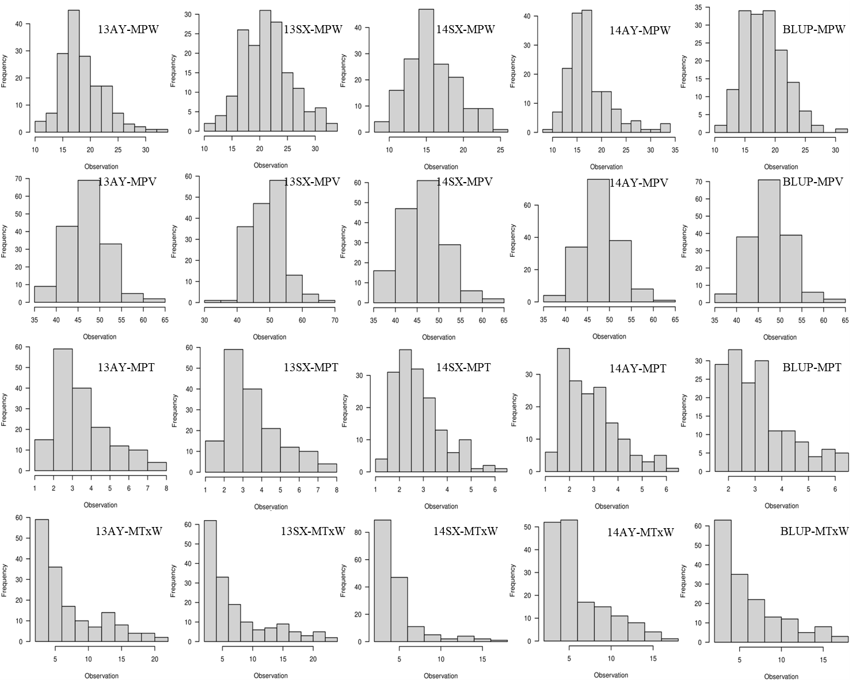

Supplement: Supplementary file 1 [file genes-14-01816-s001.zip › Figure S1.tif]

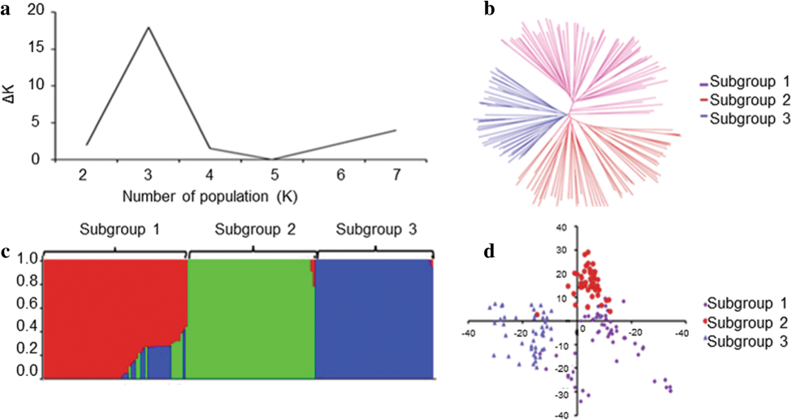

Supplement: Supplementary file 1 [file genes-14-01816-s001.zip › Figure S2.tif]

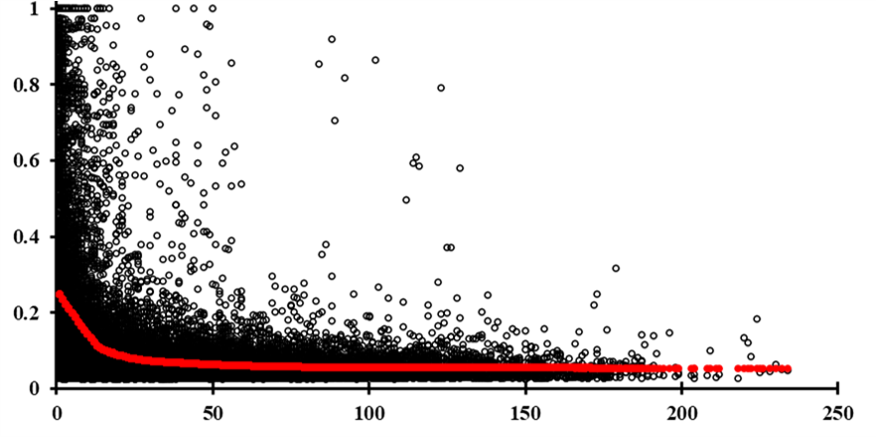

Supplement: Supplementary file 1 [file genes-14-01816-s001.zip › Figure S3.tif]

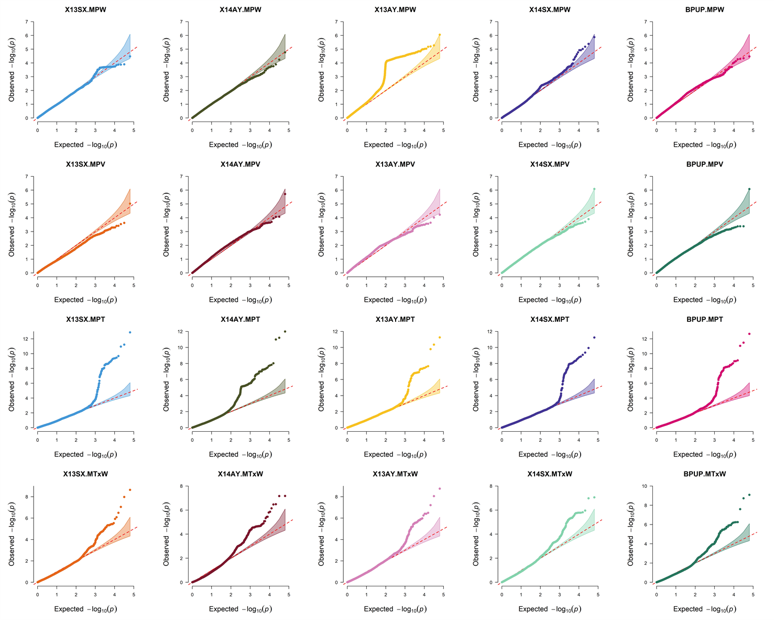

Supplement: Supplementary file 1 [file genes-14-01816-s001.zip › Figure S4.tif]
